# Dietary diversity, multidimensional gait characteristics, and related biomarkers in older adults: a wearable sensors study

**DOI:** 10.1016/j.jnha.2026.100845

**Published:** 2026-04-16

**Authors:** Chi Zhang, Yushan Zhang, Zehong Huo, Yingqi Zhao, Chuhao Zhou, Hong Shi, Ji Shen, Changzheng Yuan, Ping Zeng

**Affiliations:** a Department of Basic Innovation Research, Beijing Hospital, National Center for Gerontology, National Clinical Research Center for Gerontology, The Key Laboratory of Geriatrics of NHC, Beijing Key Laboratory of Aging Mechanism and Intervention Research on Aging-Related Diseases, Institute of Geriatric Medicine, Chinese Academy of Medical Sciences, Beijing, China; b Department of Geriatrics, Beijing Hospital, National Center for Gerontology, National Clinical Research Center for Gerontology, The Key Laboratory of Geriatrics of NHC, Institute of Geriatric Medicine, Chinese Academy of Medical Sciences, Beijing, China; c School of Public Health, the Second Affiliated Hospital, Zhejiang University School of Medicine, Hangzhou, China; d Department of Nutrition, Harvard T. H. Chan School of Public Health, Boston, MA

**Keywords:** Dietary diversity, Gait vigor, Gait stability, Wearable device, Older adults

## Abstract

**Background:**

Dietary diversity plays a crucial role in maintaining physical function. This study explored the association and potential mechanisms between dietary diversity and gait characteristics measured by wearable devices in older adults.

**Methods:**

This cross-sectional study included 861 older adults aged 60 years or above. Dietary diversity score (DDS) was assessed using a standard food frequency questionnaire. A multi-sensor gait system was used to measure periodic, kinetic, and spatiotemporal gait parameters during a 12-meter walking test. The coefficient of variation (CV) was calculated for each parameter to assess gait stability. Multivariable linear regression models were conducted to examine the relationship between DDS and gait parameters, adjusting for demographics, lifestyle factors, cognitive function, and comorbidities.

**Results:**

Participants had a mean age of 70.25 ± 6.19 years, with 58.30% being female. After adjusting for all covariates, each 1-SD increase in DDS was positively associated with Z-scores of landing control force (β = 0.072, SE = 0.033, *P* = 0.033), foot-off angle (β = 0.076, SE = 0.033, *P* = 0.021), gait speed (β = 0.086, SE = 0.033, *P* = 0.008), step length (β = 0.068, SE = 0.031, *P* = 0.032), and stride length (β = 0.078, SE = 0.033, *P* = 0.013). Furthermore, higher DDS was negatively associated with the CVs of initial limb support time, step time, stride time, ground reaction force, landing control force, foot-off angle, gait speed, and step length (all *P* < 0.05). We also identified biomarkers simultaneously related to both DDS and gait characteristics, including albumin, leptin, myostatin, brain-derived neurotrophic factor, insulin-like growth factor-1, high-sensitivity C-reactive protein, interleukin-6, and glutathione reductase.

**Conclusion:**

Higher DDS is associated with superior kinetic and spatiotemporal gait vigor performance and enhanced gait stability. Pathways involving nutritional status, energy metabolism, inflammatory regulation, antioxidant defense, and neural function may underpin this association.

## 1 Introduction

Gait performance serves as a well-established marker of overall health status in older adults, with its decline signaling increased risks of falls, functional disability, and mortality [[1], [2], [3]]. While slower gait speed represents a key indicator of frailty and sarcopenia, traditional single-parameter assessments may have limitations in capturing the full complexity of mobility decline [4,5]. Digital health technologies, particularly multi-sensor wearable gait analyzers, have transformed this field by enabling more detailed and precise assessment of gait characteristics far beyond speed alone. Unlike conventional stopwatch-based tests, which rely on subjective observation, wearable sensors enable continuous acquisition of periodic, kinetic, and spatiotemporal parameters. These real-time captured data make it feasible to monitor subtle alterations in gait vigor and stability, potentially facilitating earlier identification of functional decline and fall risk [6].

A multidimensional approach to gait analysis is essential for unraveling the intricacies of mobility in older adults. However, existing literature remains largely limited to descriptive analyses, with inadequate focus on how key modifiable lifestyle factors are mechanistically associated with refined gait metrics, especially nutritional patterns. Dietary diversity serves as a practical indicator of nutrient adequacy and is relevant for maintaining physiological homeostasis [7]. It is also a key dimension in the comprehensive assessment of dietary quality. Previous studies have shown that maintaining a diverse dietary intake is related to healthier skeletal muscle status and a lower risk of disability [8,9]. Similarly, extensive research has established positive correlations between dietary diversity and global physical performance measures including grip strength and usual walking speed [[9], [10], [11]]. A diverse diet may support gait function by providing essential substrates for muscle maintenance and energy metabolism. Furthermore, balanced nutrition can benefit multiple physiological function simultaneously by regulating energy metabolism, modulating systemic inflammation and oxidative stress, and supporting neurotrophic processes involved in motor control [[12], [13], [14]].

Of particular interest is the regulation of gait stability, a metric commonly quantified by measuring the variation between successive steps during walking tasks. A low gait variation generally reflects stable walking rhythm, whereas elevated variation values may indicate instability or reduced sensorimotor integration. Gait stability is likely influenced by the underlying physiological environment. Better nutritional status enhances stability by supporting neuromuscular coupling and mitigating metabolic stressors. Conversely, limited dietary variety might promote inflammatory or catabolic states that compromise the fine coordination required for steady gait [13,15].

Despite these established physiological interconnections, few studies have examined the potential mechanisms linking dietary diversity to gait characteristics. In the current study, we aimed to investigate the relationship between dietary diversity score (DDS) and multidimensional gait parameters measured by wearable devices in older adults. We further explored the underlying biological mechanisms which may explain the relationship between dietary diversity and better gait performance.

## 2 Methods

### 2.1 Study population

From February to August 2023, 937 older adults aged over 60 years were recruited from five communities in Beijing. The inclusion criteria were defined as follows: ① aged 60 years or above; ② clear consciousness with no communication obstacles when interacting with investigators; ③ voluntary participation in the study and willingness to cooperate with the completion of questionnaires and physical assessments. Among them, 55 participants were excluded due to activity limitations that prevented completion of the wearable gait assessment. After further excluding 21 participants with missing data on DDS or covariates, a total of 861 individuals were included in the primary association analysis. After excluding 209 participants who did not provide blood samples, a total of 652 individuals were included in the subsequent biomarker analysis. The study was approved by the Ethics Committee of Beijing Hospital (No. 2022BJYYEC-263-02), and written informed consent was obtained from all participants. The flow chart of sample recruitment was presented in Supplementary Fig. S1.

### 2.2 Sample size calculation

This was an exploratory study, and no previous research has reported the association between DDS and multidimensional gait parameters measured by wearable sensors. The effect size was therefore determined based on the results of prior studies investigating the correlation between DDS and gait speed. Sample size calculation was performed via the F-test for multiple linear regression using G*Power 3.1 software with the following parameters. The effect size f^2^ was approximately 0.015, which was converted from the predetermined correlation coefficient (set to 0.12) for the association between DDS and gait speed based on previous studies [9,16]. A total of 11 independent variables were included in the model, consisting of one core independent variable (DDS) and ten covariates. The two-sided significance level (α) was set at 0.05 and the statistical power (1-β) at 0.90. Calculation results indicated that the minimum sample size required for this study was 748 participants. Considering potential missing data and sample exclusion for sensitivity analyses during the research process, 861 participants were recruited and finally included in the study, which met the statistical power requirements for all analyses.

### 2.3 Assessment of DDS

During face-to-face surveys, trained nurses used a standard food frequency questionnaire to assess participants' consumption frequency of eight food groups (meat, eggs, fish, milk, legumes, yogurt, vegetables, and fruits). Each food group was scored as follows: 1 = never consume, 2 = occasionally consume, 3 = often consume, 4 = almost daily consume. The total score ranged from 8 to 32, with a higher score indicating better dietary diversity. The food frequency scale is culturally adapted to the Chinese population with good applicability for older adults, and has been validated to be significantly associated with major health outcomes including all-cause mortality in previous studies [17,18].

### 2.4 Gait measurements

**Fig. 1:**
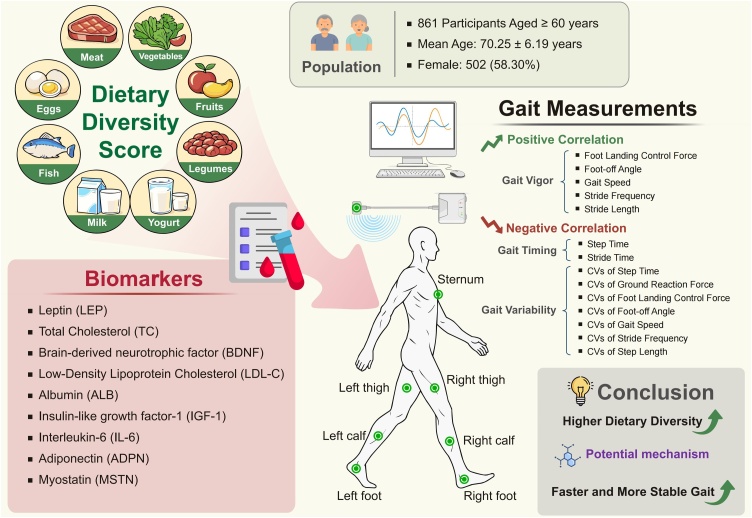
Study design and procedure for measuring gait characteristics using wearable sensors.

Gait performance was assessed using the IDEEA system (MiniSun LLC, USA), a device whose accuracy and test-retest reliability have been established in prior studies [19,20]. With seven 3D sensors attached to the sternum and bilateral lower limbs, participants were instructed to walk 12 meters on level ground at their habitual, self-selected speed without stopping. Prior to the walking test and formal data collection, participants were instructed to stand still in a neutral upright stance for 5 seconds to complete static posture initialization and ensure the stability of signal acquisition. The IDEEA system is integrated with an automated algorithm that standardizes gait parameters by excluding the gait initiation/acceleration and termination/deceleration phases, and then calculates the overall average values of gait parameters based on the valid steady-state walking segment. From the recorded biomechanical signals, a comprehensive set of 15 gait parameters was extracted and calculated using ActView software. These metrics covered three domains including periodic (double-limb support time, initial limb support time, terminal limb support time, swing time, step time, stride time), kinetic (thigh acceleration, thigh swing work, ground reaction force, landing control force, foot-off angle), and spatiotemporal (gait speed, stride frequency, step length, stride length) parameters. Gait stability was assessed by calculating the coefficient of variation (CV), defined as the ratio of the standard deviation to the mean. A higher CV value indicates greater step-to-step variability and poorer gait stability. The procedure for measuring gait characteristics based on wearable sensors is illustrated in Fig. 1.

### 2.5 Blood biomarker examination

To explore potential underlying mechanisms, a panel of circulating biomarkers mechanistically associated with DDS was detected among 652 participants who concurrently provided blood samples. These biomarkers encompassed glycolipid metabolism indicators including total cholesterol (TC), triglycerides (TG), high-density lipoprotein cholesterol (HDL-C), low-density lipoprotein cholesterol (LDL-C), and fasting plasma glucose (FPG); nutritional indicators like albumin (ALB); energy metabolism regulators such as leptin and adiponectin; neural modulation markers such as brain-derived neurotrophic factor (BDNF) and insulin-like growth factor-1 (IGF-1); renal function markers including creatinine (CREA) and uric acid (UA); skeletal muscle function-related factors including 25-hydroxyvitamin D3 [25(OH)D_3_], irisin, and myostatin; inflammatory mediators including high-sensitivity C-reactive protein (hs-CRP), interleukin-6 (IL-6), tumor necrosis factor alpha (TNF-α), and interleukin-1 beta (IL-1β); as well as oxidative stress indicators including malondialdehyde (MDA), superoxide dismutase (SOD), glutathione peroxidase (GSH-Px), and glutathione reductase (GR). Biochemical indicators were measured using a fully automated biochemical analyzer, while the other biomarkers were quantified either by RayPlex methods [21,22] or enzyme-linked immunosorbent assay (ELISA), following a unified protocol. The inter-assay and intra-assay CVs for these analytes ranged from 1.9% to 7.1% and 2.6%–8.6%, respectively.

### 2.6 Covariates

Based on previous literature [4,9,23], this study incorporated multiple covariates. Demographic characteristics (age, sex, ethnicity, and marital status) were collected through face-to-face questionnaires. Current smoking was defined as smoking at least one cigarette per day for more than six months; participants were categorized as current smokers or non-smokers (including former smokers and never smokers). Alcohol consumption was defined as drinking alcoholic beverages at least once a week for more than six months; participants were categorized as current drinkers or non-drinkers. Regular exercise was assessed referring to the physical activity item of the Fried frailty scale, with the binary classification criteria as follows: participants with weekly energy expenditure <383 kcal (equivalent to approximately 2.5 h of walking) for males and <270 kcal (equivalent to approximately 2 h of walking) for females were defined as non-regular exercisers, while those meeting or exceeding the above thresholds were defined as regular exercisers. Height and weight were measured to calculate body mass index (BMI). Cognitive function was assessed using the Mini-Mental State Examination (MMSE). Comorbidities were evaluated using the Charlson comorbidity index, which includes 25 chronic conditions [24].

### 2.7 Statistical analysis

Continuous variables with normal distribution are presented as mean ± standard deviation (M ± SD), while non-normally distributed continuous variables are described as median (interquartile range) [M (P_25_, P_75_)]. Categorical variables are expressed as numbers (percentages) [n (%)]. Spearman's correlation coefficient was used for correlation analysis between variables. Multivariable linear regression models were employed to analyze the associations between DDS and gait vigor parameters, as well as their CVs. Age, sex, ethnicity, marital status, smoking status, alcohol consumption, regular exercise, BMI, cognitive function, and comorbidities were sequentially included in the models. In the regression analysis, one gait parameter was introduced as the dependent variable each time. Multicollinearity among independent variables was assessed using variance inflation factor (VIF) and tolerance. No significant multicollinearity was observed, with all VIF values <3 (range: 1.012–1.390) and tolerance values >0.7 (range: 0.719–0.988), meeting the assumptions of linear regression (Supplementary Table S1). The normality of regression standardized residuals was assessed using histograms of regression standardized residuals and normal P-P plots of regression standardized residuals. All residuals showed an approximately normal distribution with a mean close to 0, and the residual points in the P-P plots were approximately distributed along the diagonal, satisfying the normality assumption **(**Supplementary Figure S2–S5). The homoscedasticity of residuals was verified using scatterplots of regression standardized residuals against standardized predicted values. Residual points were randomly and evenly distributed near the zero line without obvious trends, confirming the homoscedasticity assumption **(**Supplementary Figure S6–S7). Considering the differences in dimensions and value ranges among gait vigor parameters, each vigor parameter was standardized, and the Z-scores were incorporated into the linear regression. The CVs of gait parameters were included as raw values. Since no explicit hypotheses exist regarding the associations between DDS and wearable device-derived gait parameters, this study was designed as an exploratory investigation to identify potential correlations. The primary analytical objective was to systematically identify potential correlations between DDS and comprehensive gait characteristics in community-dwelling older adults, and to verify the independence of these associations via sequentially adjusted multivariable linear regression models. Secondarily, as an exploratory aim, we sought to further explore the underlying biological mechanisms linking DDS and gait performance. Intercorrelation analyses were conducted to visualize and explore the interrelationships between DDS, gait parameters, and circulating biomarkers. Consistent with methodological recommendations for exploratory research, we prioritized reducing Type II error risk to avoid overlooking meaningful associations [25,26]. Thus, no multiple testing correction was applied in the multivariable linear regression analyses.

In addition, to test the stability of the main results, the following sensitivity analyses were performed: (1) excluding 24 participants with a MMSE score < 21; (2) excluding 43 participants with stroke; (3) re-conducting the multivariable linear regression among 652 participants who provided blood samples; (4) incorporating the originally measured gait parameters as dependent variables into the model for multivariable linear regression analysis; (5) excluding participants with a BMI ≥ 30 kg/m². All statistical analyses were performed using R software (version 4.4.3), with a *P*-value < 0.05 considered statistically significant.

## 3 Results

### 3.1 Sample characteristics

The mean age of all participants was 70.25 ± 6.19 years, and 58.30% were female. The mean DDS score was 24.85 ± 3.15. Table 1 summarizes the demographic information, health status, and gait characteristics of 861 older adults.

**Table 1:** Demographic characteristics and gait parameters of 861 older adults.

Sample characteristics	n = 861
Age (m ± SD, years)	70.25 ± 6.19
Female [n (%)]	502(58.30)
Han ethnicity [n (%)]	810(94.07)
Married and living together [n (%)]	718(83.39)
BMI (m ± SD, kg/m^2^)	24.86 ± 3.45
Current Smoking [n (%)]	105(12.19)
Current alcohol consumption [n (%)]	99(11.50)
Regular exercise [n (%)]	735(85.37)
MMSE (m ± SD)	27.39 ± 2.65
Comorbidity [n (%)]	
0	137(15.91)
1	205(23.81)
2	185(21.49)
≥3	334(38.79)
Dietary diversity score (m ± SD)	24.85 ± 3.15
**Gait vigor parameters** (m ± SD)	
Double-limb support time (ms)	414.02 ± 66.39
Initial limb support time (ms)	132.76 ± 16.88
Terminal limb support time (ms)	153.46 ± 35.38
Swing time (ms)	427.61 ± 65.54
Step time (ms)	597.36 ± 115.75
Stride time (s)	1.23 ± 0.23
Thigh acceleration (G)	1.15 ± 0.41
Thigh swing work (G)	0.60 ± 0.21
Ground reaction force (G)	1.21 ± 0.41
Landing control force (G)	2.87 ± 0.98
Foot-off angle (°)	25.37 ± 12.07
Gait speed (m/min)	57.41 ± 13.92
Stride frequency (steps/min)	110.11 ± 15.47
Step length (m)	0.55 ± 0.08
Stride length (m)	1.02 ± 0.16
**Gait stability parameters** [M (P_25_, P_75_)]	
Double-limb support time (CV)	0.27(0.16,0.43)
Initial limb support time (CV)	0.22(0.17,0.31)
Terminal limb support time (CV)	0.47(0.31,0.65)
Swing time (CV)	0.26(0.15,0.42)
Step time (CV)	0.31(0.15,0.49)
Stride time (CV)	0.33(0.22,0.41)
Thigh acceleration (CV)	0.41(0.32,0.54)
Thigh swing work (CV)	0.39(0.32,0.47)
Ground reaction force (CV)	0.37(0.32,0.45)
Landing control force (CV)	0.35(0.29,0.43)
Foot-off angle (CV)	0.66(0.54,0.85)
Gait speed (CV)	0.32(0.23,0.45)
Stride frequency (CV)	0.19(0.12,0.26)
Step length (CV)	0.17(0.14,0.21)
Stride length (CV)	0.29(0.23,0.37)

**Notes:** m ± SD, mean ± standard deviation; BMI, Body mass index; MMSE, Mini-Mental State Examination; CV, Coefficient of variation.

### 3.2 Correlation between DDS and gait parameters

**Fig. 2:**
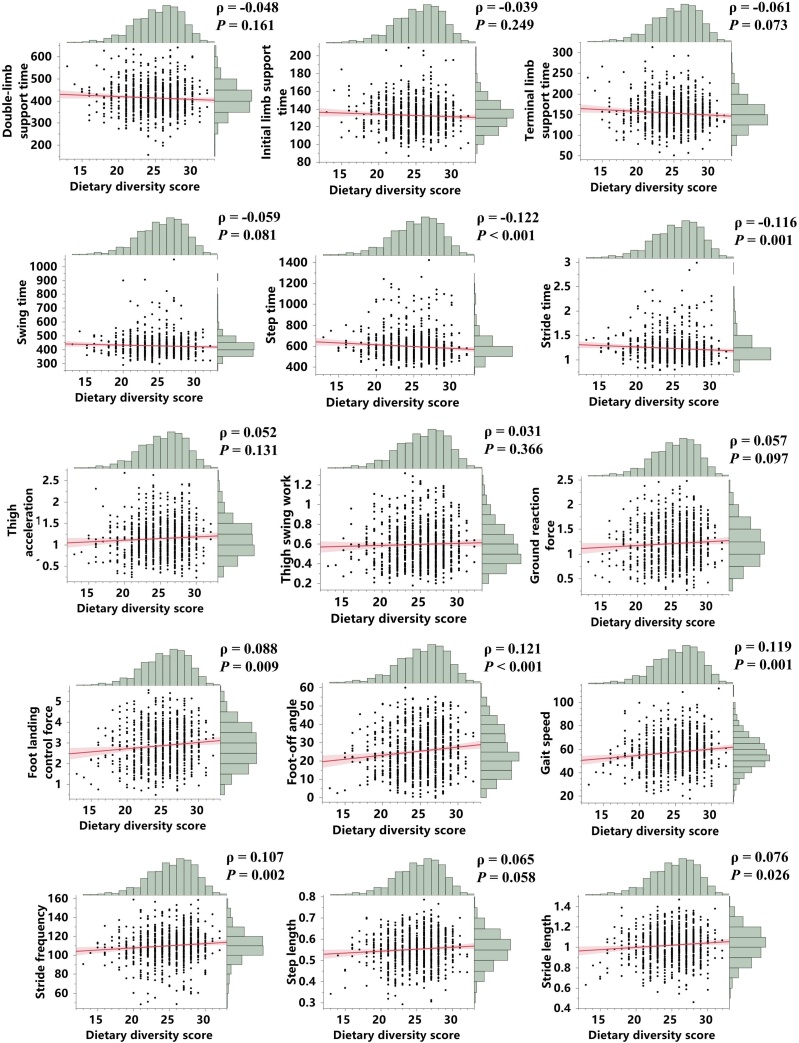
Correlations between DDS and 15 gait vigor parameters.

**Fig. 3:**
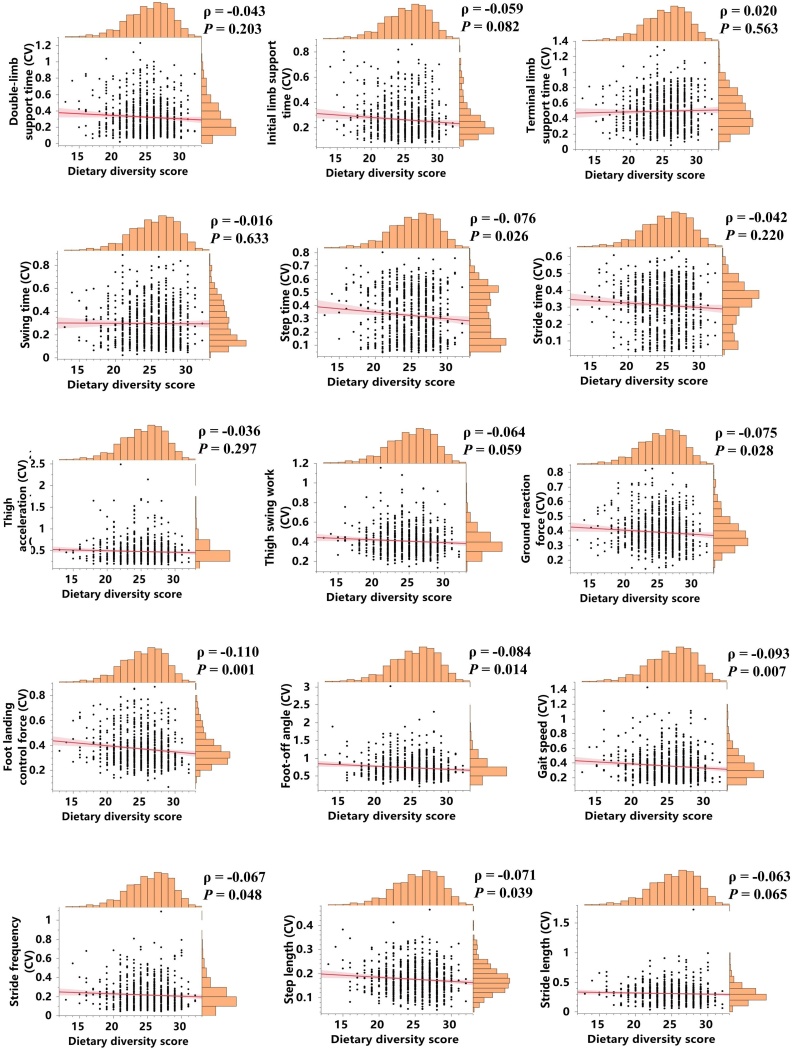
Correlations between DDS and the CVs of 15 gait parameters.

Spearman correlation analyses between DDS and gait vigor parameters are presented in Fig. 2. DDS was mildly positively correlated with landing control force (*ρ* = 0.088, *P* = 0.009), foot-off angle (*ρ* = 0.121, *P* < 0.001), gait speed (*ρ* = 0.119, *P* = 0.001), stride frequency (*ρ* = 0.107, *P* = 0.009), and stride length (*ρ* = 0.076, *P* = 0.026). While DDS was negatively mildly correlated with step time (*ρ* = −0.122, *P* < 0.001), and stride time (*ρ* = −0.116, *P* = 0.001). As shown in Fig. 3, DDS was weakly negatively associated with the CVs of step time (*ρ* = −0.076, *P* = 0.026), ground reaction force (*ρ* = −0.075, *P* = 0.028), landing control force (*ρ* = −0.110, *P* = 0.001), foot-off angle (*ρ* = −0.084, *P* = 0.014), gait speed (*ρ* = −0.093, *P* = 0.007), stride frequency (*ρ* = −0.067, *P* = 0.048), and step length (*ρ* = −0.071, *P* = 0.039). We also separately analyzed the relationships between the consumption frequency of specific food categories and gait parameters as well as their variability. Among all eight food types, consumption frequency of meat, fish, legumes, and fruit showed significant correlations with gait vigor parameters (Supplementary Figure S8). Furthermore, meat, fish, and milk were significantly negatively correlated with the CVs of specific parameters, including initial limb support time, step time, thigh acceleration, thigh swing work, ground reaction force, landing control force, gait speed, stride frequency, and stride length (Supplementary Figure S9).

### 3.3 Multivariable regression analyses between DDS and gait parameters

Multivariable linear regression analyses of the association between DDS and gait parameters (Z score) are shown in Table 2. After adjusting for all covariates, DDS (per 1-SD increase) was positively associated with landing control force (β = 0.072, *P* = 0.033), foot-off angle (β = 0.076, *P* = 0.021), gait speed (β = 0.086, *P* = 0.008), step length (β = 0.068, *P* = 0.032), and stride length (β = 0.078, *P* = 0.013). Furthermore, DDS was negatively associated with the CVs of initial limb support time, step time, stride time, ground reaction force, landing control force, foot-off angle, gait speed, and step length (all *P* < 0.05). The association remained stable across the three different adjusted models.

**Table 2:** Multivariable linear regression analysis of the association between DDS and gait parameters.

Dependent variables	Model 1			Model 2			Model 3		
	β	SE	*P*-value	β	SE	*P*-value	β	SE	*P*-value
**Gait vigor**									
Double-limb support time (Z score)	−0.031	0.034	0.078	−0.033	0.032	0.306	−0.011	0.034	0.749
Initial limb support time (Z score)	−0.051	0.034	0.132	−0.038	0.033	0.258	−0.038	0.036	0.285
Terminal limb support time (Z score)	−0.078	0.034	0.023	−0.067	0.033	0.048	−0.034	0.035	0.326
Swing time (Z score)	−0.048	0.035	0.161	−0.022	0.033	0.503	−0.017	0.035	0.622
Step time (Z score)	−0.086	0.034	0.011	−0.071	0.034	0.036	−0.052	0.035	0.134
Stride time (Z score)	−0.075	0.034	0.027	−0.057	0.034	0.089	−0.038	0.035	0.275
Thigh acceleration (Z score)	0.059	0.034	0.082	−0.064	0.034	0.061	0.048	0.035	0.168
Thigh swing work (Z score)	0.032	0.034	0.355	0.037	0.032	0.251	0.017	0.034	0.619
Ground reaction force (Z score)	0.061	0.032	0.078	0.066	0.031	0.039	0.041	0.033	0.219
Landing control force (Z score)	0.097	0.033	0.004	0.096	0.032	0.003	0.072	0.033	0.033
Foot-off angle (Z score)	0.116	0.034	0.001	0.096	0.032	0.003	0.076	0.033	0.021
Gait speed (Z score)	0.122	0.034	<0.001	0.121	0.032	<0.001	0.086	0.032	0.008
Stride frequency (Z score)	0.092	0.034	0.007	0.069	0.033	0.036	0.039	0.034	0.258
Step length (Z score)	0.076	0.034	0.025	0.095	0.029	0.001	0.068	0.031	0.032
Stride length (Z score)	0.088	0.033	0.009	0.103	0.030	0.001	0.078	0.032	0.013
**Gait stability**									
Double-limb support time (CV)	−0.013	0.007	0.058	−0.012	0.007	0.073	−0.011	0.007	0.151
Initial limb support time (CV)	−0.012	0.005	0.009	−0.012	0.004	0.013	−0.012	0.005	0.014
Terminal limb support time (CV)	0.006	0.007	0.442	0.008	0.007	0.267	0.008	0.008	0.343
Swing time (CV)	−0.001	0.006	0.854	0.001	0.006	0.967	−0.001	0.006	0.906
Step time (CV)	−0.016	0.006	0.009	−0.016	0.006	0.011	−0.017	0.006	0.008
Stride time (CV)	−0.008	0.004	0.059	−0.009	0.004	0.054	−0.011	0.005	0.028
Thigh acceleration (CV)	−0.011	0.008	0.179	−0.009	0.008	0.259	−0.009	0.008	0.323
Thigh swing work (CV)	−0.009	0.004	0.036	−0.007	0.004	0.093	−0.004	0.005	0.332
Ground reaction force (CV)	−0.009	0.004	0.022	−0.007	0.003	0.048	−0.008	0.004	0.043
Landing control force (CV)	−0.016	0.004	<0.001	−0.015	0.004	0.001	−0.014	0.005	0.002
Foot-off angle (CV)	−0.027	0.009	0.005	−0.022	0.009	0.021	−0.019	0.009	0.041
Gait speed (CV)	−0.017	0.006	0.004	−0.017	0.006	0.006	−0.017	0.006	0.009
Stride frequency (CV)	−0.008	0.004	0.078	−0.007	0.004	0.121	−0.008	0.005	0.094
Step length (CV)	−0.005	0.002	0.006	−0.005	0.002	0.017	−0.005	0.002	0.043
Stride length (CV)	−0.006	0.005	0.217	−0.005	0.005	0.313	−0.006	0.005	0.229

Notes: CV, coefficient of variation; SE, standard error.

Model 1: Unadjusted;

Model 2: Adjusted for age and sex;

Model 3: Additionally adjusted for ethnicity, marital status, smoking, alcohol consumption, regular exercise, BMI, cognitive function, and comorbidities.

### 3.4 Potential mechanisms

**Fig. 4:**
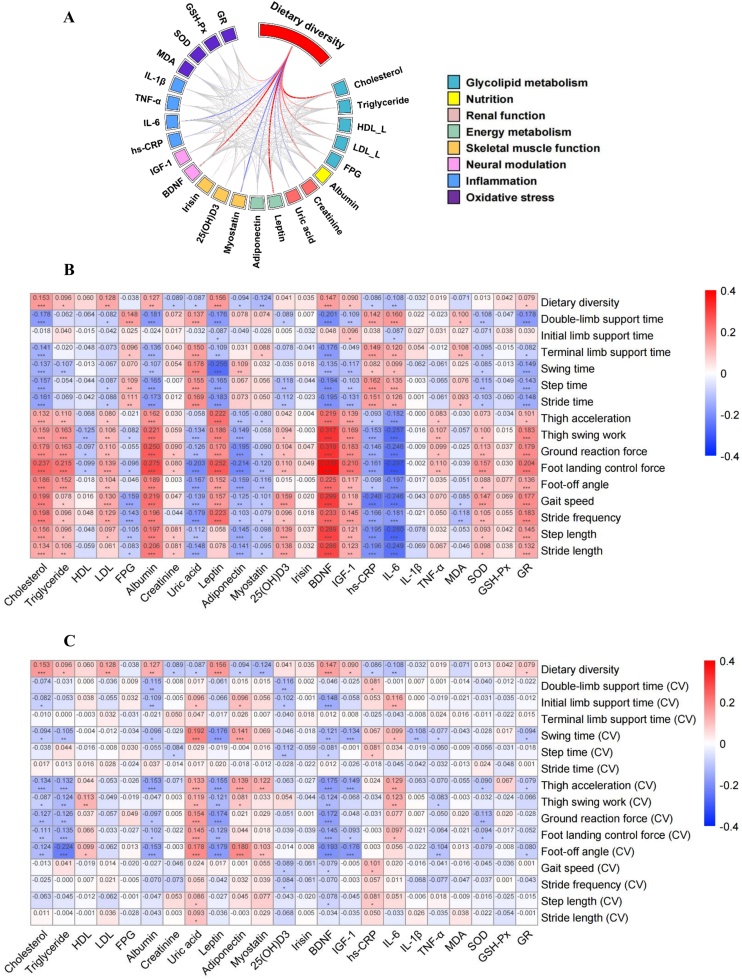
Potential mechanisms of the association between DDS and gait parameters (Fig. A: Correlations between DDS and related biomarkers; Fig. B: Correlations of biomarkers with gait vigor parameters; Fig. C: Correlations of biomarkers with gait stability parameter.).

To explore potential mechanisms through which DDS may influence gait performance, we further conducted correlation analyses among a series of related biomarkers. As shown in Fig. 4, DDS showed significant positive correlations with TC (*ρ* = 0.153, *P* < 0.001), LDL-C (*ρ* = 0.128, *P* < 0.001), albumin (*ρ* = 0.127, *P* < 0.001), leptin (*ρ* = 0.156, *P* < 0.001), BDNF (*ρ* = 0.147, *P* < 0.001), IGF-1 (*ρ* = 0.090, *P* = 0.003), and GR (*ρ* = 0.079, *P* = 0.016). Conversely, significant negative correlations were observed with creatinine (*ρ* = −0.089, *P* = 0.008), UA (ρ = −0.087, *P* = 0.011), adiponectin (*ρ* = −0.094, *P* = 0.002), myostatin (*ρ* = −0.124, *P* < 0.001), hs-CRP (*ρ* = −0.086, *P* = 0.004), and IL-6 (*ρ* = −0.108, *P* < 0.001).

TC, albumin, leptin, BDNF, SOD, 25(OH)D_3_, IGF-1, and GR demonstrated negative correlations with periodic parameters while showing positive correlations with kinetic and spatiotemporal parameters. Conversely, UA, adiponectin, myostatin, hs-CRP, and IL-6 exhibited negative correlations with most kinetic and spatiotemporal parameters. Among DDS-related biomarkers, albumin, leptin, BDNF, and IGF-1 were simultaneously negatively correlated with the CVs of kinetic gait parameters. In contrast, UA, adiponectin, myostatin, and IL-6 showed positive correlations with the variability of specific gait parameters, including thigh acceleration, ground reaction force, landing control force, and foot-off angle.

### 3.5 Sensitivity analyses

We conducted sensitivity analyses to assess the potential bias introduced by severe physical and cognitive impairments. After excluding 24 participants with a MMSE score <21, DDS maintained significant positive associations with landing control force, foot-off angle, gait speed, step length, and stride length. Moreover, DDS remained negatively associated with the CVs of six parameters including initial limb support time, step time, stride time, ground reaction force, landing control force, and gait speed (Supplementary Table S2). In addition, after excluding 43 individuals with a history of stroke, the associations of DDS with gait vigor and stability metrics remained consistent with the main results (Supplementary Table S3). Finally, we also re-conducted multiple linear regression analyses among 652 participants included in biomarker analysis. The association patterns between DDS and gait parameters persisted, although statistical significance was marginally attenuated (Supplementary Table S4). When the originally observed gait parameters were used to replace the Z-scores, the correlation characteristics between DDS and gait parameters remained unchanged (Supplementary Table S5). Additionally, after excluding individuals with excessively high BMI, DDS maintained significant positive associations with landing control force, foot-off angle, gait speed, step length, and stride length, and remained significantly negatively associated with the CVs of initial limb support time, step time, landing control force, foot-off angle, and gait speed in the fully adjusted model (Supplementary Table S6).

## 4 Discussion

Using wearable-sensor devices, our study utilized high-dimensional gait features to provide more systematic biomechanical information on gait mobility than walk pace alone. We observed that even after adjusting for demographic factors and various health statuses, dietary diversity was positively associated with kinetic and spatiotemporal gait vigor performance, and was negatively related to gait variability.

Our observations are generally consistent with existing literature indicating that high dietary variation may protect against physical decline. Previous epidemiological studies have linked higher dietary diversity to reduced risks of sarcopenia, frailty, and functional disability [23,27,28]. However, most prior research has treated gait as a unidimensional functional output focusing primarily on walking speed [16,29]. By incorporating periodic, kinetic, and spatiotemporal measurements, the present study attempts to advance this understanding by proposing that dietary diversity helps maintain the stability of gait parameters, which may be a sensitive predictor of falls and mobility impairment. Specifically, our fully adjusted multivariable linear regression analyses revealed that each 1-SD increase in DDS was significantly and positively associated with the Z-scores of landing control force (β = 0.072, *P* = 0.033), foot-off angle (β = 0.076, *P* = 0.021), gait speed (β = 0.086, *P* = 0.008), step length (β = 0.068, *P* = 0.032) and stride length (β = 0.078, *P* = 0.013) in older adults, even after rigorous adjustment for a comprehensive set of confounders including demographic characteristics, lifestyle factors, cognitive function and comorbidities. These core regression results demonstrated the independent and robust association between higher dietary diversity and better kinetic as well as spatiotemporal gait vigor performance, and the consistent direction and statistical significance of the regression coefficients across the sequentially adjusted models further verified the stability of this association. We observed that dietary diversity was not significantly associated with periodic parameters, a finding that may stem from periodic gait parameters being more strongly modulated by non-nutritional factors such as age-related neuromuscular aging and inherent motor control rhythms rather than dietary intake. These non-significant associations imply that the regulatory effect of dietary diversity on gait performance is specific to the kinetic and spatiotemporal dimensions of walking, rather than exerting a broad influence on all gait metrics.

The potential impact of dietary diversity on gait is rooted in the cumulative effect of macro- and micronutrients provided by various food groups. As a key dimension of dietary quality, the DDS effectively reflects the balanced and sufficient food intake that is fundamental to optimal nutrient supply in older adults. These nutrients likely interact synergistically to modulate the synthesis and metabolism of key biomarkers, thereby creating a systemic environment that supports musculoskeletal function. Building upon our observed associations between dietary diversity and gait parameters, we further explored the underlying biological mechanisms including nutritional, metabolic, and myogenic pathways. First, DDS demonstrated a significant positive correlation with albumin, a well-established marker of nutritional status. Albumin plays an essential role in maintaining oncotic pressure and transporting nutrients, thereby supporting skeletal muscle integrity. Higher DDS implies a more comprehensive nutrient intake, which may elevate circulating albumin levels and consequently preserve muscle mass and function, forming the physiological foundation for enhanced gait vigor [30,31]. Second, we observed that DDS was positively correlated with leptin but negatively correlated with adiponectin. Leptin regulates energy expenditure and glucose metabolism, ensuring sufficient energy supply for physical activity [32]. Meanwhile, elevated adiponectin levels have been associated with frailty and muscle wasting in older adults, possibly reflecting a compensatory response to metabolic stress [33]. Thus, the observed negative correlation between DDS and adiponectin may indicate improved metabolic homeostasis, potentially reflecting a transition from catabolic states characterized by muscle breakdown toward anabolic states that favor muscle preservation [34]. Third, a key pathway involves the direct regulation of skeletal muscle. DDS was positively associated with 25(OH)D_3_, while negatively correlated with myostatin. Dietary intake of foods rich in vitamin D, such as fatty fish, egg yolks, and fortified dairy products, contributes to the synthesis and activation of 25(OH)D_3_, which is crucial for calcium homeostasis and muscle contractility. In contrast, myostatin functions as a potent negative regulator of muscle growth [35]. The capacity of a diverse diet to simultaneously upregulate vitamin D and suppress myostatin highlights a dual mechanism that promotes muscle maintenance and reduces atrophy, thereby directly enhancing the kinetic and spatiotemporal dimensions of gait performance [36,37].

The continuous and stable execution of walking relies heavily on the coordinated cooperation between the nervous system and skeletal muscles [38]. Beyond muscular and metabolic factors, our results indicated that DDS might improve gait performance through neuroprotective and immunomodulatory mechanisms. The significant positive correlations between DDS and both BDNF and IGF-1 underscore a neurotrophic pathway. BDNF and IGF-1 cooperate to promote neuronal survival, synaptic plasticity, and neuromuscular junction integrity [39,40]. This neurotrophic support is critical for the central nervous system's ability to coordinate complex motor tasks, explaining the improved gait stability associated with higher dietary diversity. DDS was also associated with lower inflammatory markers such as hs-CRP and IL-6. Chronic inflammation is a well-established driver of sarcopenia and physical frailty [41,42]. By providing anti-inflammatory nutrients, a high DDS may dampen systemic inflammation, thereby protecting muscle tissue from inflammatory degradation. Finally, the positive association with GR suggests an enhanced antioxidant defense. DDS may increase the activity of key antioxidant enzymes, thereby helping to mitigate oxidative stress. This process creates a protective effect against musculoskeletal aging and preserves motor units [43]. It should also be noted that our biomarker analyses only identified correlational associations rather than causal relationships between dietary diversity, circulating biomarkers, and gait parameters. Future longitudinal studies or rigorously designed dietary intervention trials are needed to validate the causal pathways linking these three factors in older adults. Notably, we did not assess muscle strength, muscle mass, frailty status, sarcopenia, balance function, fatigue, depressive symptoms, or specific nutrient intakes, all of which may act as key mediators or confounders in the relationships between dietary diversity, biomarkers and gait parameters and merit further investigation. Additionally, these unmeasured factors limit our ability to fully elucidate the complex biological pathways underlying the observed associations. Future studies are also warranted to perform stratified analyses by different frailty conditions and verify the robustness of the observed associations.

A novel finding of this study is the independent association between dietary diversity and gait stability in older adults. In our fully adjusted multivariable linear regression models, we found that higher DDS was significantly negatively associated with the CVs of multiple key gait parameters, including initial limb support time, step time, stride time, ground reaction force, landing control force, foot-off angle, gait speed, and step length. This association persisted after rigorous adjustment for a wide range of confounding factors, and remained stable across all sequential adjustment models and sensitivity analyses. In particular, neurotrophic and anabolic markers such as albumin, leptin, BDNF, and IGF-1 were negatively correlated with gait variability. Mechanistically, BDNF and IGF-1 enhance neuromuscular transmission and sensorimotor integration, facilitating synchronized muscle activation [44,45]. This improved coordination reduces the fluctuation and asymmetry of kinetic parameters such as landing control force and foot-off angle during the gait cycle. Conversely, catabolic and inflammatory markers such as UA, adiponectin, myostatin, and IL-6 were positively correlated with gait variability. Higher IL-6 and myostatin may disrupt muscle firing patterns and exacerbate neuroinflammation, leading to irregular gait rhythms [46]. Similarly, abnormally high UA levels may be related to metabolic and neurological disturbances that impair fine motor control [47]. Overall, these results suggest that DDS promotes gait stability not through a single pathway, but by orchestrating a physiological environment that favors neuromuscular coordination while suppressing destabilizing factors. Although wearable sensors allowed us to monitor the average performance of gait characteristics in the present study, the slight weight of the sensor device may have caused the collected gait parameters to deviate slightly from real-world unassisted walking, such as a marginally lower measured gait speed than the actual level. Future research should leverage complementary technologies such as optical motion capture and pressure mats to further enrich gait assessment indicators, particularly for characterizing the differences between various swing and stance phases of the gait cycle.

Despite the findings of this study, several limitations should be acknowledged. First, the cross-sectional design precludes causal inference regarding the relationship between dietary diversity and gait performance. Future longitudinal studies are warranted to clarify the temporal relationship between dietary diversity and gait changes in older adults. Second, older adults who are able to complete wearable walking tests may have better physical function and greater capacity to acquire diverse foods, thereby inflating the observed association between DDS and gait metrics. Third, dietary assessment relied on food frequency questionnaires which may cause recall bias. We did not quantify the intake of energy or specific nutrients in the present study, and future investigations should further explore the potential mediating roles of various nutrients in the association between dietary diversity and gait characteristics. Fourth, while we adjusted for numerous health-related confounders, residual confounding from unmeasured variables, including genetic susceptibility and environmental factors, was not considered. Fifth, this study treated dietary diversity as an overall indicator of healthy eating behavior. Although we separately analyzed the intake from specific food sources, future research is needed to explore the relationship between diverse dietary patterns and gait characteristics. Finally, potential false-positive associations cannot be fully ruled out in this study. Our preliminary findings require cautious interpretation prior to independent replication in external larger samples.

## 5 Conclusion

This study suggests that higher DDS is associated with better gait vigor and stability in community-dwelling older adults. The potential mechanisms may involve a complex interplay of multiple intertwined pathways, including nutritional status, energy metabolism, inflammatory regulation, antioxidant defense, and neural function. These findings indicate that maintaining dietary diversity could serve as a practical and modifiable strategy to enhance gait mobility in older individuals.

## CRediT authorship contribution statement

C Zhang analyzed the data and wrote the manuscript; Y Zhang and Z Huo collected and interpreted the data; Y Zhao, C Zhou and H Shi contributed to critical revision of the article; J Shen and C Yuan contributed to methodology and supervision; J Shen and P Zeng participated in the study design and contributed to critical revision of the article. All authors have read and agreed to the published version of the article.

## Consent for publication

Not applicable.

## Ethics approval and consent to participate

The study protocol was approved by the Ethics Committee of Beijing Hospital (Approval No. 2022BJYYEC-263-02), and written informed consent was obtained from all participants.

## Declaration of Generative AI and AI-assisted technologies in the writing processs

The authors only used AI to correct the grammar in some sentences.

## Funding

This study is supported by the National High Level Hospital Clinical Research Funding (BJ-2023-072,BJ-2024-175,BJ-2025-253,BJ-2025-240,BJ-2024-219,BJ-2022-133) and the National Science and Technology Major Project for the Prevention and Treatment of Cancer, Cardiovascular and Cerebrovascular, Respiratory and Metabolic Diseases (2024ZD0524100,2024ZD0524104).

## Availability of data and materials

The datasets analyzed during the current study are available from the corresponding author on reasonable request.

## Declaration of competing interest

The authors declare that they have no competing interests.
