# Supplementary material for: Dietary diversity, multidimensional gait characteristics, and related biomarkers in older adults: a wearable sensors study
Source: J Nutr Health Aging. 2026 Apr 16;30(6):100845. doi: 10.1016/j.jnha.2026.100845 (PMC13098583; doi:10.1016/j.jnha.2026.100845)
Supplement: Supplementary file 1 [file mmc1.docx]

**Supplementary materials**


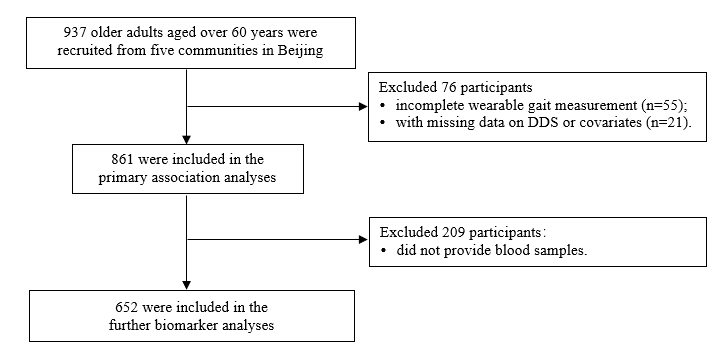


**Supplementary figure 1.** Flowchart of participant recruitment

**Supplementary table 1.** Variance inflation factor (VIF) and tolerance of all independent variables

| **Independent variables** | **VIF** | **Tolerance** |
| --- | --- | --- |
| Dietary diversity (per 1-SD) | 1.085 | 0.921 |
| Age | 1.153 | 0.867 |
| Sex | 1.390 | 0.719 |
| Ethnicity | 1.012 | 0.988 |
| Marriage | 1.099 | 0.910 |
| Smoking | 1.382 | 0.724 |
| Alcohol consumption | 1.338 | 0.747 |
| Regular exercise | 1.033 | 0.968 |
| Body mass index | 1.033 | 0.968 |
| Cognitive function | 1.081 | 0.925 |
| Comorbidities | 1.108 | 0.903 |


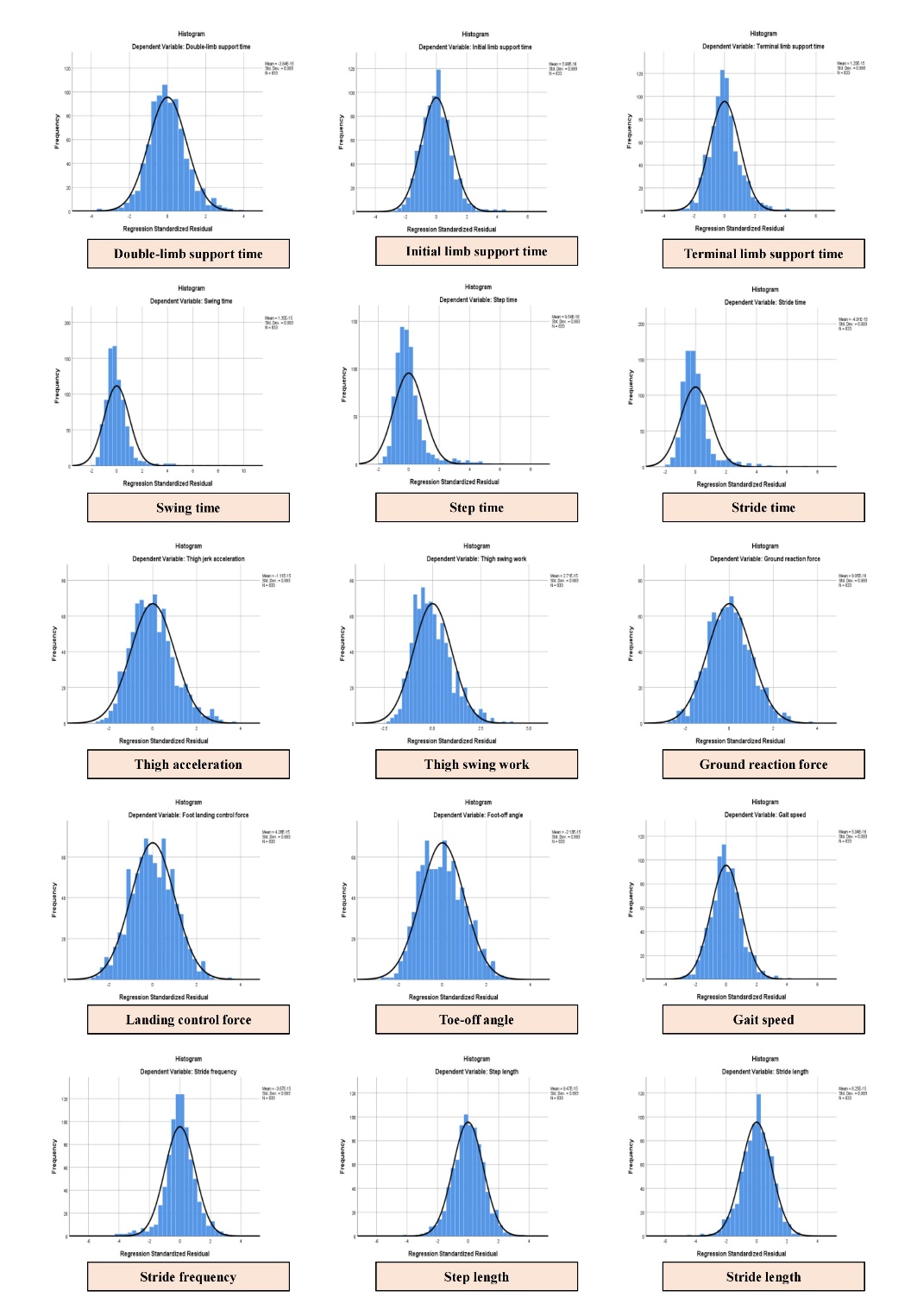


**Supplementary figure 2.** Histograms of regression standardized residuals for 15 gait vigor parameters in the fully adjusted model.


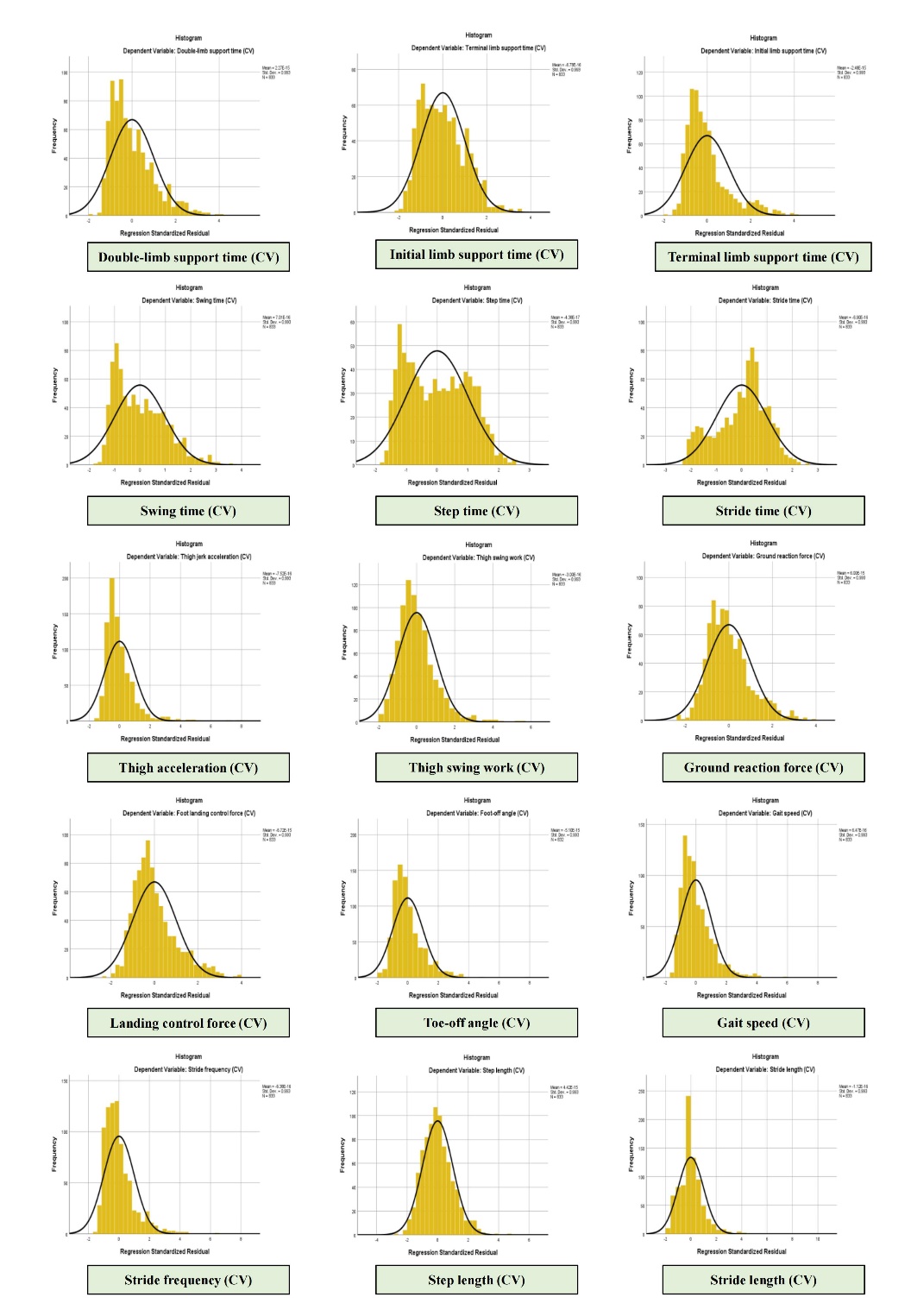


**Supplementary figure 3.** Histograms of regression standardized residuals for 15 gait stability parameters in the fully adjusted model.


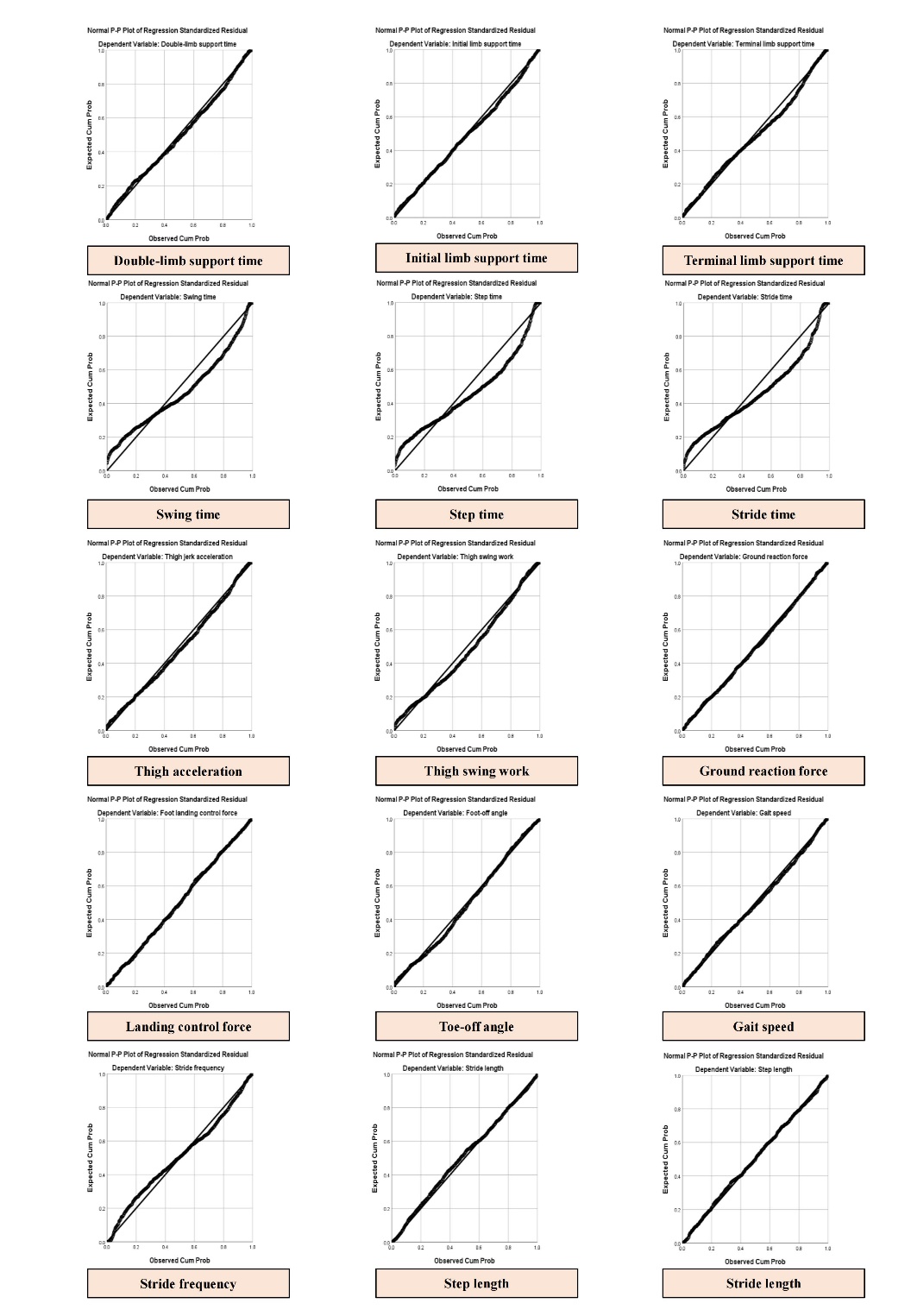


**Supplementary figure 4.** Normal P-P plots of regression standardized residuals for 15 gait vigor parameters in the fully adjusted model.


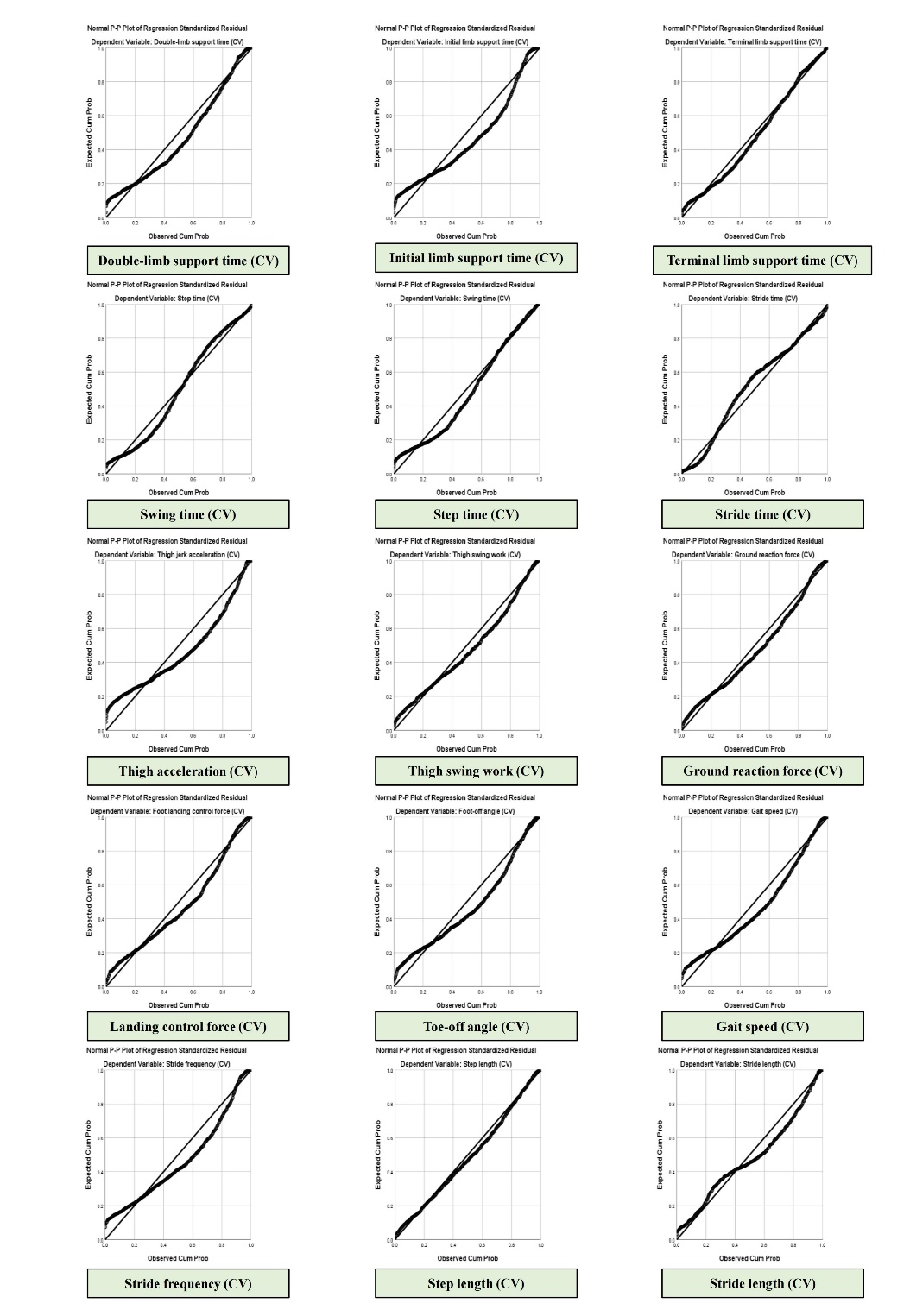


**Supplementary figure 5.** Normal P-P plots of regression standardized residuals for 15 gait stability parameters in the fully adjusted model.


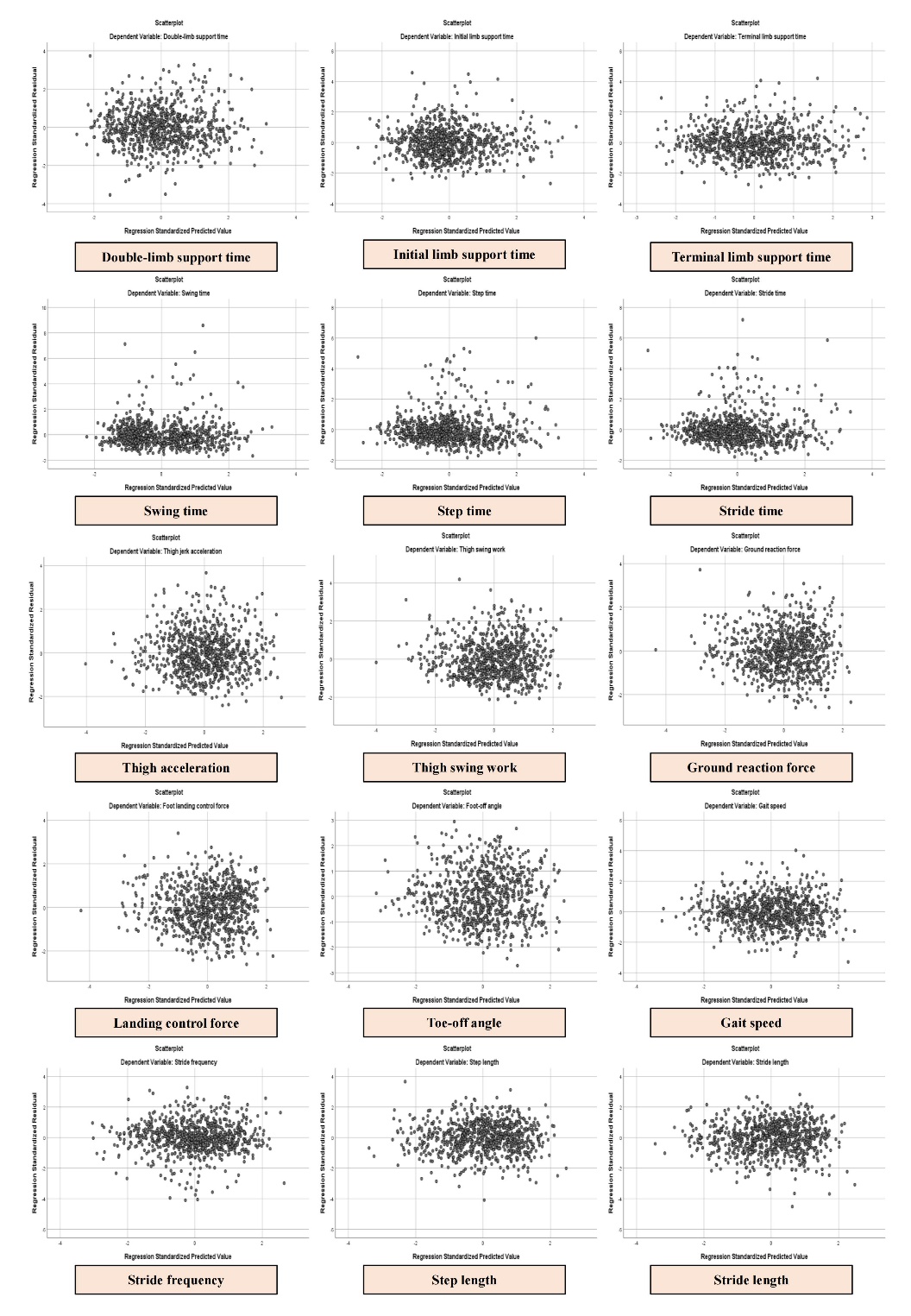


**Supplementary figure 6.** scatterplots of regression standardized residuals against standardized predicted values for 15 gait vigor parameters in the fully adjusted model.


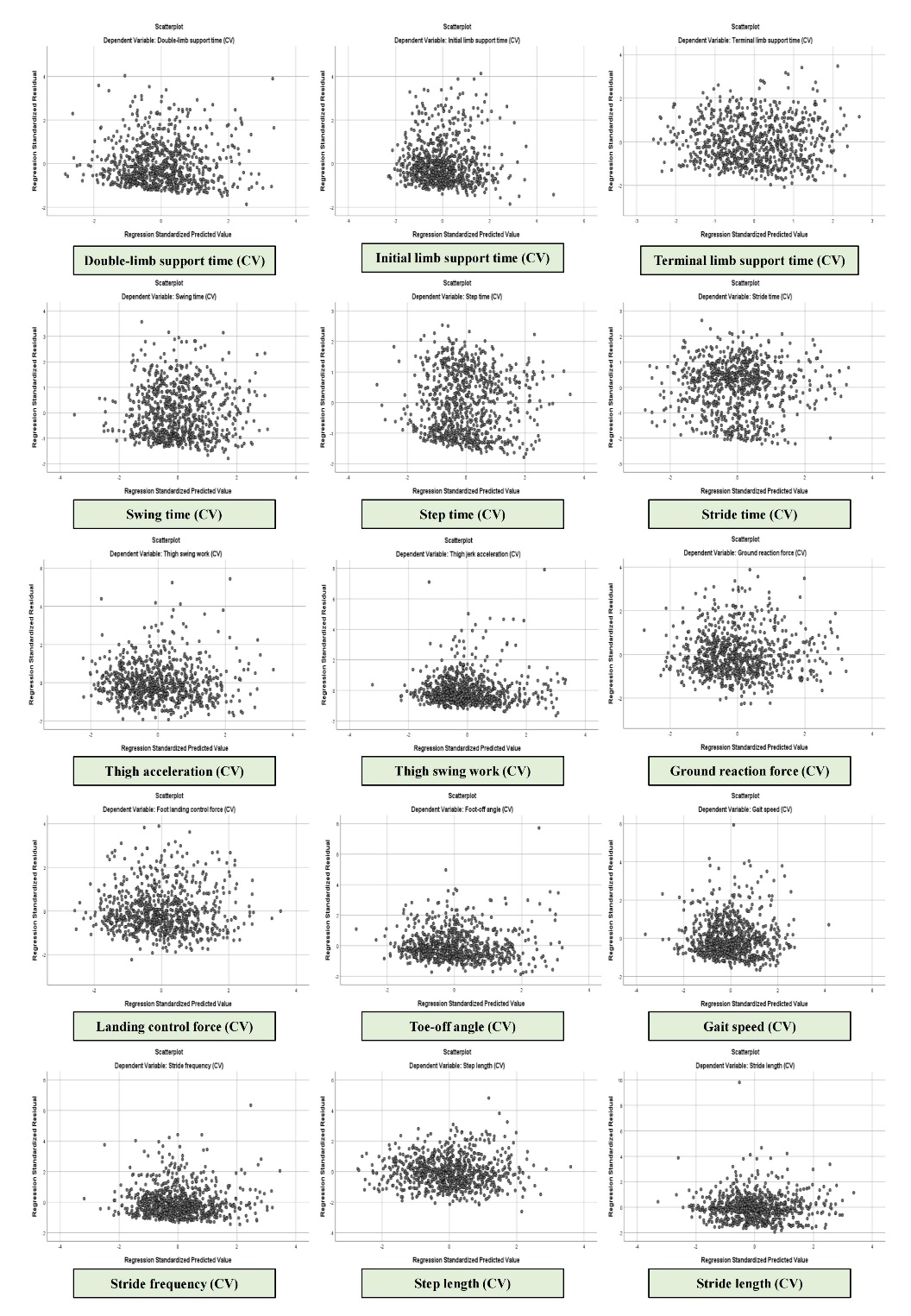


**Supplementary figure 7.** Scatterplots of regression standardized residuals against standardized predicted values for 15 gait stability parameters in the fully adjusted model.


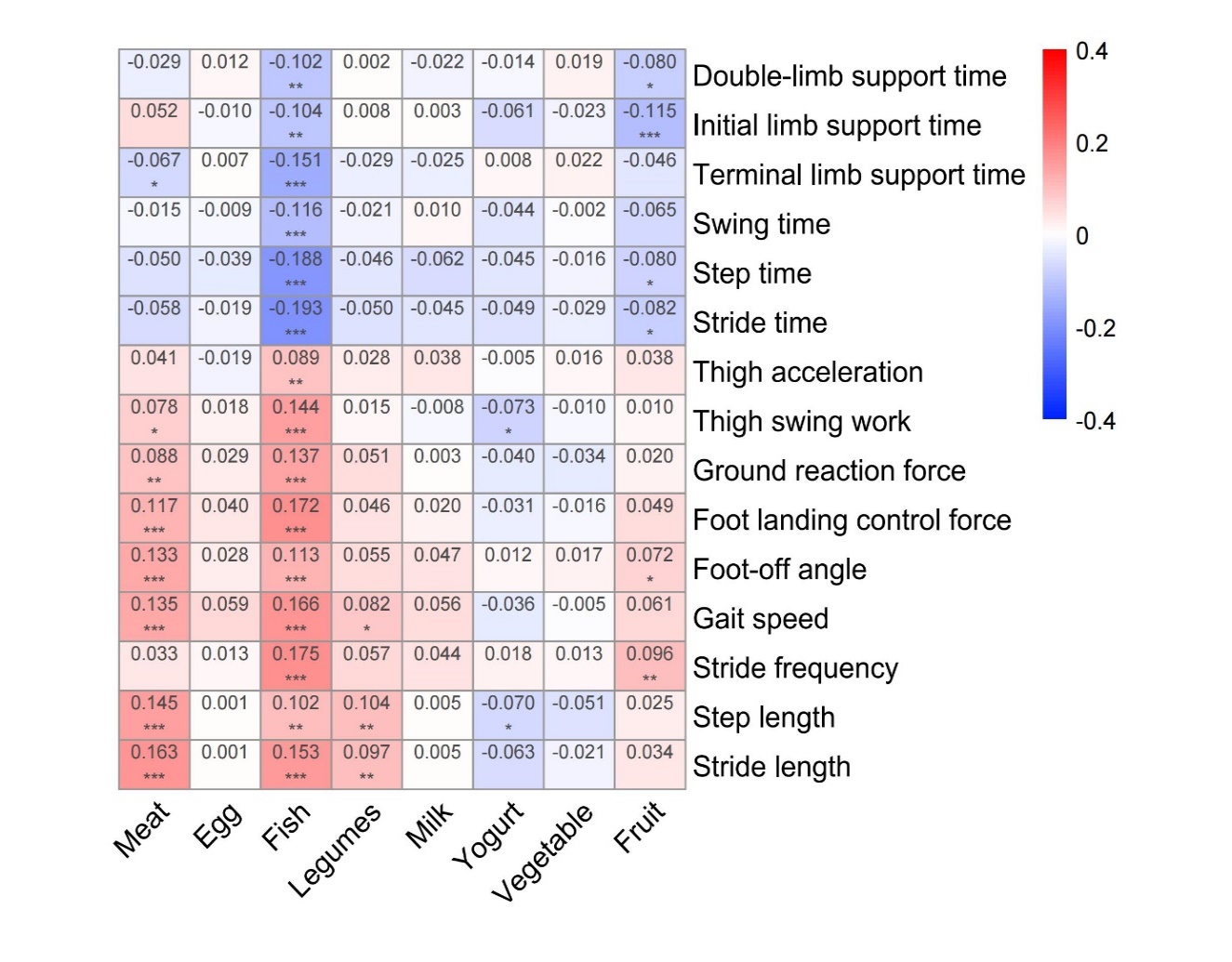


**Supplementary figure 8.** Correlations of specific food intake frequency with gait vitality parameters


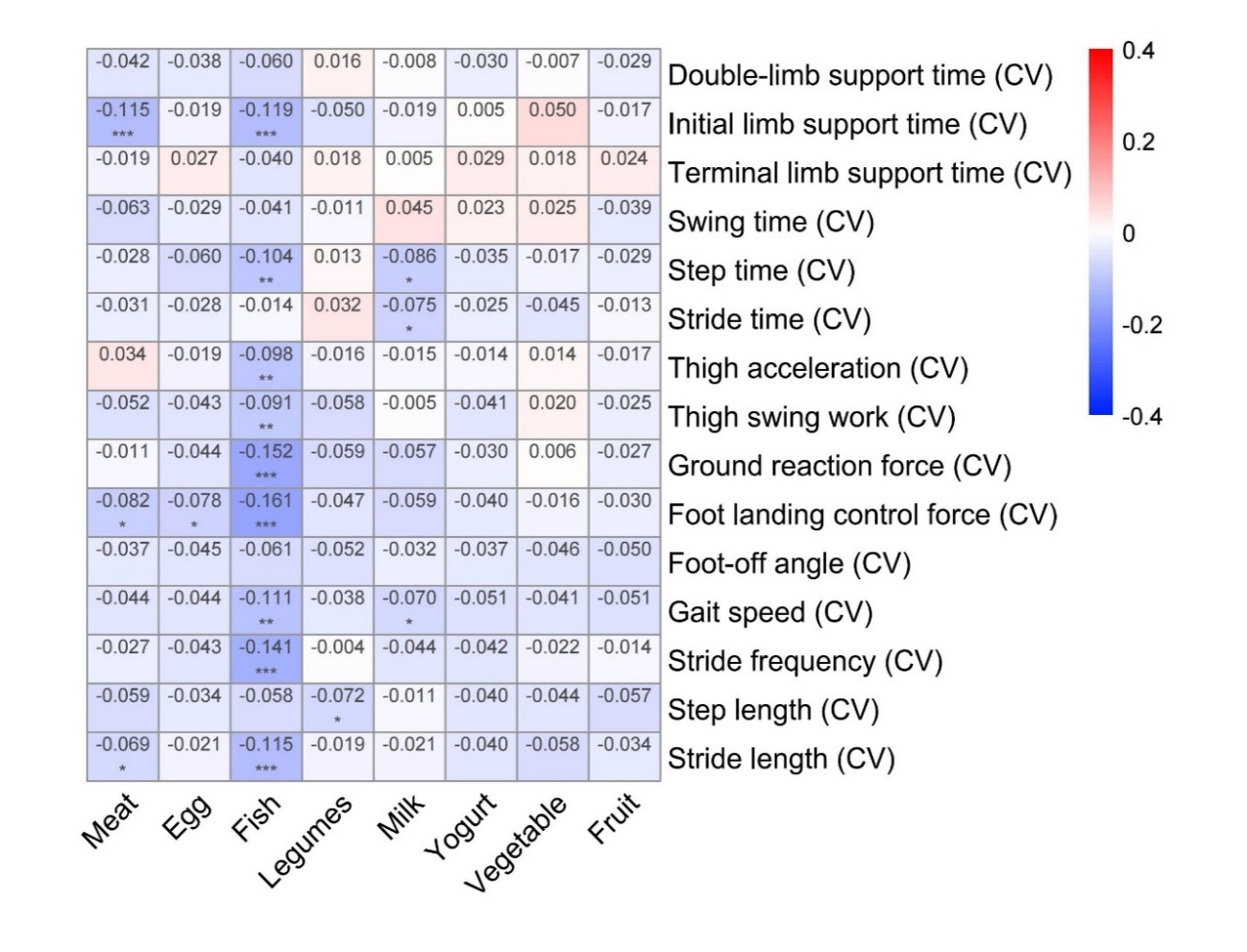


**Supplementary figure 9.** Correlations of specific food intake frequency with gait stability parameters

**Supplementary table 2.** Multivariable linear regression of the association between DDS and gait parameters after excluding 24 participants with MMSE < 21

| Dependent variables | Fully adjusted model | | |
| --- | --- | --- | --- |
|  | β | SE | *P*-value |
| Gait vigor |  |  |  |
| Double-limb support time (Z score) | 0.001 | 0.035 | 0.988 |
| Initial limb support time (Z score) | -0.031 | 0.036 | 0.389 |
| Terminal limb support time (Z score) | -0.035 | 0.036 | 0.336 |
| Swing time (Z score) | -0.011 | 0.035 | 0.749 |
| Step time (Z score) | -0.057 | 0.035 | 0.105 |
| Stride time (Z score) | -0.042 | 0.036 | 0.237 |
| Thigh acceleration (Z score) | 0.041 | 0.037 | 0.261 |
| Thigh swing work (Z score) | 0.021 | 0.035 | 0.557 |
| Ground reaction force (Z score) | 0.412 | 0.034 | 0.232 |
| Landing control force (Z score) | 0.071 | 0.034 | 0.040 |
| Foot-off angle (Z score) | 0.077 | 0.034 | 0.024 |
| Gait speed (Z score) | 0.093 | 0.033 | 0.005 |
| Stride frequency (Z score) | 0.049 | 0.035 | 0.159 |
| Step length (Z score) | 0.064 | 0.031 | 0.041 |
| Stride length (Z score) | 0.072 | 0.032 | 0.026 |
| Gait stability |  |  |  |
| Double-limb support time (CV) | -0.011 | 0.007 | 0.168 |
| Initial limb support time (CV) | -0.012 | 0.005 | 0.015 |
| Terminal limb support time (CV) | 0.008 | 0.008 | 0.319 |
| Swing time (CV) | 0.001 | 0.006 | 0.839 |
| Step time (CV) | -0.019 | 0.006 | 0.006 |
| Stride time (CV) | -0.011 | 0.005 | 0.036 |
| Thigh acceleration (CV) | -0.011 | 0.009 | 0.235 |
| Thigh swing work (CV) | -0.005 | 0.008 | 0.311 |
| Ground reaction force (CV) | -0.008 | 0.004 | 0.054 |
| Landing control force (CV) | -0.015 | 0.005 | 0.002 |
| Foot-off angle (CV) | -0.018 | 0.011 | 0.077 |
| Gait speed (CV) | -0.017 | 0.007 | 0.011 |
| Stride frequency (CV) | -0.007 | 0.005 | 0.155 |
| Step length (CV) | -0.003 | 0.002 | 0.156 |
| Stride length (CV) | -0.004 | 0.006 | 0.501 |

Adjusted for age, sex, ethnicity, education level, marriage, smoking, alcohol consumption, regular exercise, BMI, cognitive function, and comorbidities.

**Supplementary table 3.** Multivariable linear regression of the association between DDS and gait parameters after excluding 43 participants with stroke

| Dependent variables | Fully adjusted model | | |
| --- | --- | --- | --- |
|  | β | SE | *P*-value |
| Gait vigor |  |  |  |
| Double-limb support time (Z score) | 0.011 | 0.035 | 0.739 |
| Initial limb support time (Z score) | -0.038 | 0.037 | 0.306 |
| Terminal limb support time (Z score) | -0.027 | 0.036 | 0.441 |
| Swing time (Z score) | -0.001 | 0.036 | 0.976 |
| Step time (Z score) | -0.041 | 0.036 | 0.261 |
| Stride time (Z score) | -0.027 | 0.036 | 0.454 |
| Thigh acceleration (Z score) | 0.026 | 0.037 | 0.473 |
| Thigh swing work (Z score) | 0.006 | 0.035 | 0.857 |
| Ground reaction force (Z score) | 0.031 | 0.034 | 0.375 |
| Landing control force (Z score) | 0.055 | 0.034 | 0.111 |
| Foot-off angle (Z score) | 0.071 | 0.034 | 0.040 |
| Gait speed (Z score) | 0.076 | 0.033 | 0.022 |
| Stride frequency (Z score) | 0.022 | 0.035 | 0.542 |
| Step length (Z score) | 0.052 | 0.031 | 0.082 |
| Stride length (Z score) | 0.072 | 0.032 | 0.025 |
| Gait stability |  |  |  |
| Double-limb support time (CV) | -0.012 | 0.008 | 0.109 |
| Initial limb support time (CV) | -0.014 | 0.005 | 0.005 |
| Terminal limb support time (CV) | 0.006 | 0.008 | 0.458 |
| Swing time (CV) | 0.001 | 0.006 | 0.981 |
| Step time (CV) | -0.019 | 0.007 | 0.004 |
| Stride time (CV) | -0.011 | 0.005 | 0.033 |
| Thigh acceleration (CV) | -0.010 | 0.009 | 0.271 |
| Thigh swing work (CV) | -0.004 | 0.005 | 0.423 |
| Ground reaction force (CV) | -0.007 | 0.004 | 0.071 |
| Landing control force (CV) | -0.014 | 0.005 | 0.004 |
| Foot-off angle (CV) | -0.023 | 0.011 | 0.021 |
| Gait speed (CV) | -0.019 | 0.007 | 0.006 |
| Stride frequency (CV) | -0.008 | 0.005 | 0.103 |
| Step length (CV) | -0.005 | 0.002 | 0.034 |
| Stride length (CV) | -0.007 | 0.005 | 0.211 |

Adjusted for age, sex, ethnicity, education level, marriage, smoking, alcohol consumption, regular exercise, BMI, cognitive function, and comorbidities.

**Supplementary table 4.** Multivariable linear regression of the association between DDS and gait parameters among 652 participants who provided blood samples

| Dependent variables | Fully adjusted model | | |
| --- | --- | --- | --- |
|  | β | SE | *P*-value |
| Gait vigor |  |  |  |
| Double-limb support time (Z score) | -0.001 | 0.037 | 0.985 |
| Initial limb support time (Z score) | -0.037 | 0.039 | 0.344 |
| Terminal limb support time (Z score) | -0.042 | 0.038 | 0.268 |
| Swing time (Z score) | -0.035 | 0.039 | 0.375 |
| Step time (Z score) | -0.053 | 0.039 | 0.171 |
| Stride time (Z score) | -0.039 | 0.038 | 0.313 |
| Thigh acceleration (Z score) | 0.057 | 0.041 | 0.161 |
| Thigh swing work (Z score) | 0.004 | 0.039 | 0.823 |
| Ground reaction force (Z score) | 0.014 | 0.037 | 0.711 |
| Landing control force (Z score) | 0.055 | 0.037 | 0.138 |
| Foot-off angle (Z score) | 0.083 | 0.037 | 0.026 |
| Gait speed (Z score) | 0.093 | 0.035 | 0.009 |
| Stride frequency (Z score) | 0.047 | 0.037 | 0.209 |
| Step length (Z score) | 0.059 | 0.031 | 0.047 |
| Stride length (Z score) | 0.075 | 0.035 | 0.037 |
| Gait stability |  |  |  |
| Double-limb support time (CV) | -0.005 | 0.008 | 0.548 |
| Initial limb support time (CV) | -0.011 | 0.004 | 0.038 |
| Terminal limb support time (CV) | 0.011 | 0.009 | 0.208 |
| Swing time (CV) | -0.002 | 0.007 | 0.779 |
| Step time (CV) | -0.013 | 0.007 | 0.087 |
| Stride time (CV) | -0.007 | 0.005 | 0.204 |
| Thigh acceleration (CV) | -0.005 | 0.009 | 0.642 |
| Thigh swing work (CV) | -0.001 | 0.005 | 0.848 |
| Ground reaction force (CV) | -0.009 | 0.004 | 0.039 |
| Landing control force (CV) | -0.014 | 0.005 | 0.011 |
| Foot-off angle (CV) | -0.017 | 0.011 | 0.122 |
| Gait speed (CV) | -0.014 | 0.006 | 0.038 |
| Stride frequency (CV) | -0.005 | 0.005 | 0.352 |
| Step length (CV) | -0.003 | 0.002 | 0.281 |
| Stride length (CV) | -0.003 | 0.006 | 0.641 |

Adjusted for age, sex, ethnicity, education level, marriage, smoking, alcohol consumption, regular exercise, BMI, cognitive function, and comorbidities.

**Supplementary table 5.** Multivariable linear regression of the association between DDS and vigor gait parameters among 861 older adults

| **Dependent variables** | **Model 1** | | | **Model 2** | | | **Model 3** | | |
| --- | --- | --- | --- | --- | --- | --- | --- | --- | --- |
|  | β | SE | *P*-value | β | SE | *P*-value | β | SE | *P*-value |
| **Gait vigor** |  |  |  |  |  |  |  |  |  |
| Double-limb support time (ms) | -3.989 | 2.251 | 0.078 | -1.975 | 2.194 | 0.368 | 0.967 | 2.281 | 0.671 |
| Initial limb support time (ms) | -0.867 | 0.575 | 0.132 | -0.675 | 0.574 | 0.269 | -0.663 | 0.602 | 0.271 |
| Terminal limb support time (ms) | -2.741 | 1.203 | 0.023 | -2.256 | 1.196 | 0.059 | -1.099 | 1.229 | 0.372 |
| Swing time (ms) | -3.423 | 2.437 | 0.161 | -1.527 | 2.285 | 0.522 | -1.115 | 2.515 | 0.657 |
| Step time (ms) | -10.902 | 4.272 | 0.011 | -8.317 | 4.185 | 0.047 | -6.264 | 4.396 | 0.153 |
| Stride time (s) | -0.019 | 0.008 | 0.027 | -0.013 | 0.008 | 0.108 | -0.009 | 0.008 | 0.308 |
| Thigh acceleration (G) | 0.024 | 0.014 | 0.082 | 0.026 | 0.014 | 0.065 | 0.019 | 0.015 | 0.178 |
| Thigh swing work (G) | 0.006 | 0.007 | 0.355 | 0.007 | 0.007 | 0.332 | 0.003 | 0.007 | 0.722 |
| Ground reaction force (G) | 0.024 | 0.013 | 0.078 | 0.023 | 0.013 | 0.067 | 0.014 | 0.013 | 0.291 |
| Landing control force (G) | 0.096 | 0.033 | 0.004 | 0.087 | 0.031 | 0.006 | 0.061 | 0.021 | 0.035 |
| Foot-off angle (°) | 1.397 | 0.409 | 0.001 | 1.072 | 0.386 | 0.006 | 0.863 | 0.402 | 0.032 |
| Gait speed (m/min) | 1.692 | 0.471 | <0.001 | 1.571 | 0.446 | 0.001 | 1.122 | 0.453 | 0.013 |
| Stride frequency (steps/min) | 1.427 | 0.525 | 0.007 | 1.009 | 0.509 | 0.048 | 0.545 | 0.526 | 0.301 |
| Step length (m) | 0.006 | 0.003 | 0.025 | 0.007 | 0.002 | 0.004 | 0.005 | 0.002 | 0.029 |
| Stride length (m) | 0.014 | 0.005 | 0.009 | 0.015 | 0.005 | 0.002 | 0.011 | 0.005 | 0.026 |

Notes: All gait parameters were included as originally observed measurements. CV, coefficient of variation; SE, standard error.

Model 1: No adjusted;

Model 2: Adjusted for age and sex;

Model 3: Additionally adjusted for ethnicity, marriage, smoking, alcohol consumption, regular exercise, BMI, cognitive function, and comorbidities.

**Supplementary table 6.** Multivariable linear regression of the association between DDS and gait parameters after excluding 55 participants with BMI ≥30 kg/m^2^

| Dependent variables | Fully adjusted model | | |
| --- | --- | --- | --- |
|  | β | SE | *P*-value |
| Gait vigor |  |  |  |
| Double-limb support time (Z score) | 0.003 | 0.036 | 0.835 |
| Initial limb support time (Z score) | -0.026 | 0.037 | 0.489 |
| Terminal limb support time (Z score) | -0.029 | 0.036 | 0.421 |
| Swing time (Z score) | -0.004 | 0.037 | 0.901 |
| Step time (Z score) | -0.039 | 0.036 | 0.281 |
| Stride time (Z score) | -0.024 | 0.036 | 0.504 |
| Thigh acceleration (Z score) | 0.047 | 0.037 | 0.201 |
| Thigh swing work (Z score) | 0.009 | 0.035 | 0.785 |
| Ground reaction force (Z score) | 0.029 | 0.034 | 0.397 |
| Landing control force (Z score) | 0.068 | 0.034 | 0.037 |
| Foot-off angle (Z score) | 0.075 | 0.034 | 0.029 |
| Gait speed (Z score) | 0.081 | 0.033 | 0.017 |
| Stride frequency (Z score) | 0.029 | 0.036 | 0.416 |
| Step length (Z score) | 0.062 | 0.031 | 0.048 |
| Stride length (Z score) | 0.069 | 0.032 | 0.035 |
| Gait stability |  |  |  |
| Double-limb support time (CV) | -0.009 | 0.007 | 0.243 |
| Initial limb support time (CV) | -0.011 | 0.005 | 0.012 |
| Terminal limb support time (CV) | 0.007 | 0.008 | 0.416 |
| Swing time (CV) | 0.002 | 0.006 | 0.773 |
| Step time (CV) | -0.016 | 0.007 | 0.016 |
| Stride time (CV) | -0.010 | 0.005 | 0.046 |
| Thigh acceleration (CV) | -0.013 | 0.009 | 0.169 |
| Thigh swing work (CV) | -0.005 | 0.005 | 0.319 |
| Ground reaction force (CV) | -0.008 | 0.004 | 0.052 |
| Landing control force (CV) | -0.014 | 0.005 | 0.003 |
| Foot-off angle (CV) | -0.018 | 0.008 | 0.031 |
| Gait speed (CV) | -0.018 | 0.007 | 0.009 |
| Stride frequency (CV) | -0.006 | 0.005 | 0.195 |
| Step length (CV) | -0.004 | 0.002 | 0.059 |
| Stride length (CV) | -0.004 | 0.006 | 0.445 |

Adjusted for age, sex, ethnicity, education level, marriage, smoking, alcohol consumption, regular exercise, BMI, cognitive function, and comorbidities.
